# Amplification of Emerging Viruses in a Bat Colony

**DOI:** 10.3201/eid1703.100526

**Published:** 2011-03

**Authors:** Jan Felix Drexler, Victor Max Corman, Tom Wegner, Adriana Fumie Tateno, Rodrigo Melim Zerbinati, Florian Gloza-Rausch, Antje Seebens, Marcel A. Müller, Christian Drosten

**Affiliations:** Author affiliations: University of Bonn Medical Centre, Bonn, Germany (J.F. Drexler, V.M. Corman, A.F. Tateno, R.M. Zerbinati, F. Gloza-Rausch, A. Seebens, M.A. Müller, C. Drosten);; University of Bonn, Bonn (T. Wegner);; Noctalis, Centre for Bat Protection and Information, Bad Segeberg, Germany (F. Gloza-Rausch, A. Seebens)

**Keywords:** Zoonoses, bats, coronavirus, astrovirus, adenovirus, viruses, zoonotic risk assessment, reservoir ecology, research

## Abstract

Bats host noteworthy viral pathogens, including coronaviruses, astroviruses, and adenoviruses. Knowledge on the ecology of reservoir-borne viruses is critical for preventive approaches against zoonotic epidemics. We studied a maternity colony of *Myotis myotis* bats in the attic of a private house in a suburban neighborhood in Rhineland-Palatinate, Germany, during 2008, 2009, and 2010. One coronavirus, 6 astroviruses, and 1 novel adenovirus were identified and monitored quantitatively. Strong and specific amplification of RNA viruses, but not of DNA viruses, occurred during colony formation and after parturition. The breeding success of the colony was significantly better in 2010 than in 2008, in spite of stronger amplification of coronaviruses and astroviruses in 2010, suggesting that these viruses had little pathogenic influence on bats. However, the general correlation of virus and bat population dynamics suggests that bats control infections similar to other mammals and that they may well experience epidemics of viruses under certain circumstances.

## Abstract 2

Concentration and prevalence of coronaviruses and astroviruses increase when bats form maternity roosts and bear young.

Bats (Chiroptera) constitute ≈20% of living mammal species and are distributed on all continents except Antarctica (*1*). Their ability to fly and migrate, as well as the large sizes of social groups, predispose them for the acquisition and maintenance of viruses (*2*). Although the ways of contact are unknown, bat-borne viruses can be passed to other mammals and cause epidemics (*2**,**3*). Several seminal studies have recently implicated bats as sources of important RNA viruses of humans and livestock, including lyssaviruses, coronaviruses (CoVs), filoviruses, henipaviruses, and astroviruses (AstVs) (*2**,**4*). DNA viruses, including herpesviruses and adenoviruses (AdVs), have also been detected in bats, although with less clear implications regarding the role of bats as sources of infection for other mammals (*5**–**8*). While most of the above-mentioned viruses are carried by tropical fruit bats (Megachiroptera), the predominant hosts of mammalian CoVs, including those related to the agent of severe acute respiratory syndrome (SARS), are insectivorous bats (Microchiroptera) that are not restricted to tropical climates (*1*). By demonstrating the presence of SARS-related CoV in Europe, we have recently shown that the geographic extent of its reservoir is much larger than that of other bat-borne viruses, including Ebola, Marburg, Nipah, and Hendra (*9*).

In spite of the potential for serious consequences of virus epidemics emerging from bats, knowledge is currently lacking on the ecology of bat-borne viruses in bat reservoirs. We do not know how viruses with human pathogenic potential are maintained in bat populations, whether and how they are amplified and controlled, and whether they cause effects on individual bats or on bat populations. The current lack of data is due to difficulties in monitoring virus populations (rather than bat populations) in sufficient density. Available studies have focused on lyssaviruses, hennipaviruses, and filoviruses, which have extremely low detection frequencies, thus causing viruses to be encountered too rarely to enable the characterization of virus frequency and concentration over time (*10**–**15*). These studies have therefore relied on antibody testing, which provides higher detection rates by making indirect and cumulative assessments of virus contact during the lifetime of bats (*10**–**15*). However, results of antibody testing fail to correlate with the current presence of virus, preventing reliable analysis of a time component.

In a recent study, we obtained preliminary statistical hints that bats were more likely to carry CoV if they were young (*16*). In adult bats, a significant risk of carrying virus was identified for lactating females (*16*). Taking these clues together, we speculated that maternity roosts, inhabited predominantly by lactating females and newborns, with few adult males (*17*), might serve as the compartment of CoV amplification within the yearly life-cycle of bats in temperate climates. We therefore investigated the patterns of maintenance and amplification of specific RNA- and DNA viruses by direct and quantitative virus detection in a maternity colony over 3 consecutive years. RNA- and DNA viruses were examined because of their different abilities to persist and to rapidly generate new variants. Viruses identified included 1 CoV, 6 different AstVs, as well as a novel bat AdV. To assess the pathogenic influence of these viruses on bats, we quantified the reproductive success of the colony over the same time period.

## Materials and Methods

### Sample Collection and Preparation

**Figure 1:**
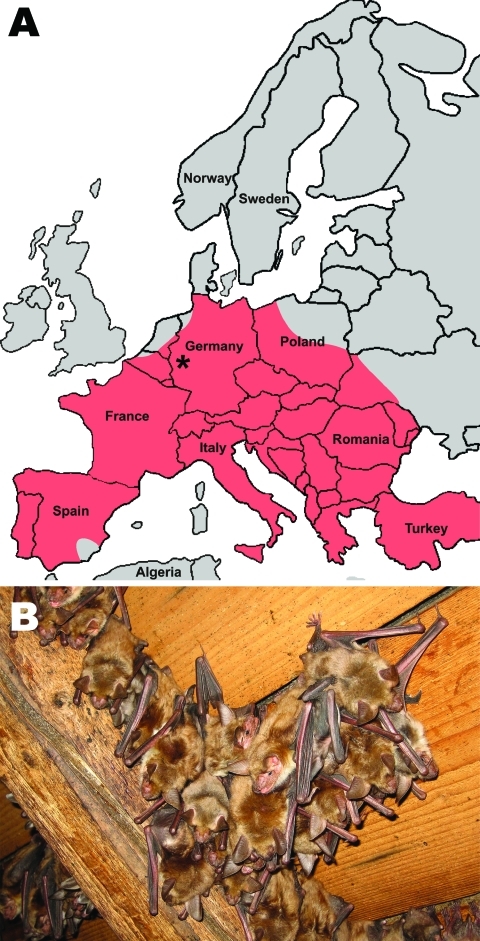
A) Location of studied maternity bat roost (indicated by asterisk) in the state of Rhineland-Palatinate, Germany (50°25′46.91′′N, 6°55′52.17′′E). Red shading indicates the distribution of the studied bat species (adapted from the IUCN Red List of Threatened Species, v. 2010; www.iucnredlist.org). B) Cluster of *Myotis myotis* female bats hanging from the roof interior**.**

Permission for this work on protected bats was obtained from the environmental protection authority (Struktur-Und Genehmigungsbehörde Nord Koblenz) of the German federal state of Rhineland-Palatinate. Sampling took place over 3 consecutive years: 2008, 2009, and 2010. The sampling site was the attic of a private house in a suburban area in the state of Rhineland-Palatinate, western Germany (Figure 1). The study did not involve any direct manipulations of bats and relied entirely on collection of fecal samples from the attic floor. Classification of bats as *Myotis myotis* was confirmed by mitochondrial DNA typing as described (*9*). Adult female bats leaving the roost were counted by trained field biologists before and after parturition. Pups were counted in the sampling site after the departure of adults. For each sampling date, plastic film was spread in the evening on the ground of a 20-m^2^ attic compartment, and fresh droppings were collected with clean disposable forks the following night. Each sample consisted of exactly 5 fecal pellets collected in proximity and added to RNAlater RNA preservative solution (QIAGEN, Hilden, Germany). The equivalent of ≈100 mg was purified by the Viral RNA kit (QIAGEN) according to manufacturer’s instructions.

### Detection and Quantification of Viral RNA/DNA

Five microliters of RNA/DNA eluate were tested by broad range reverse transcription–PCR (RT-PCR) assays for the whole subfamily *Coronavirinae* (*16*), the family *Astroviridae* (*4*), and the genus *Mastadenovirus* (*18*). Specific real-time RT-PCR oligonucleotides were designed within the initial PCR fragments (those used are shown in Table 1). All 4 described real-time RT-PCR assays showed comparable lower limits of detection in the single copy range. Twenty-five–microliter reactions used the SuperScript III PlatinumOne-Step qRT-PCR Kit (Invitrogen, Karlsruhe, Germany) for detecting CoVs and AstVs in *M. myotis* bats or the Platinum Taq DNA Polymerase Kit (Invitrogen) for detecting AdVs in *M. myotis* bats. Reactions were generally composed as follows: 400 nmol/L of the respective primers, 200 nmol/L of the respective hydrolysis probe, 0.5 µL enzyme mix or 0.1 µL Platinum Taq, 1 µg bovine serum albumin, and 5 µL RNA/DNA extract. For AdV DNA PCR, supplements of 0.2 mmol/L of each dNTP and 2.0 mmol/L of MgCL were added. Amplification involved 15 min at 55°C for reverse transcription of RNA viruses and 3 min at 95°C, followed by 45 cycles of 15 seconds at 94°C, and 25 seconds at 58°C for all viruses. Fluorescence was measured at the 58°C annealing/extension step.

**Table 1:** Real-time reverse transcription–PCR oligonucleotides used for RNA virus testing, Germany, 2008–2010*

Virus targeted	Oligonucleotide ID	Sequence, 5′ → 3′	Orientation
Coronavirus	CoV-F	CGTCTGGTGATGCTACTACTGCTT	+
	CoV-P	FAM-TGCAAATTCCGTCTTTAAT-MGBNFQ	Probe
	CoV-R	CATTGGCACTAACAGCCTGAAA	–
Astrovirus	AstVa-F	GCTTGATCCWGTCTATCATACTGATG	+
	AstVa-P	FAM-CTTTTGAGTTTGCGTATGTTCA-MGBNFQ	Probe
	AstVa-R	CACATTTTTTCCATTCTTCTTCAAG	–
	AstVb-F	TATGTACTACTGCCTTCTGGTGAAATC	+
	AstVb-P	YAK-CCCACCAAACTCGCGGGAATCCT-BBQ1	Probe
	AstVb-R	TTATCCATCGTTGTGCTCACTTG	–
Adenovirus	AdV-F	GCGGTTGCAGCTAAGATTTGT	+
	AdV-P	FAM-CCCGTGGACAAAGAAGACACCCAGTATG-BBQ1	Probe
	AdV-R	CCAGCTGGAAGCGTGTTTTAT	–

*ID, identification; CoV, coronavirus; AstV, atrovirus; AdV, adenovirus; FAM, 6-carboxyfluorescein; MGB, minor groove binder; NFQ, nonfluorescent quencher; YAK, Yakima yellow; BBQ, black berry quencher; +, positive; –, negative.

For quantification, PCR amplicons from the initial screening assay were TA cloned in a pCR 4.0 vector (Invitrogen). Plasmids were then purified and reamplified with vector-specific oligonucleotides, followed by in vitro transcription with a T7 promotor-based Megascript kit (Applied Biosystems, Darmstadt, Germany). The in vitro–transcribed RNAs or, in the case of AdVs, the photometrically quantified plasmid alone, were used as calibration standards for virus quantification in bat fecal samples, as described previously (*19*).

### In Silico Analyses

Sanger sequencing of PCR products was done by using dye terminator chemistry (Applied Biosystems). Nucleic acid alignments with prototype virus sequences were done based on amino acid code by the BLOSUM algorithm in the MEGA4 software package (www.megasoftware.net). Neighbor-joining phylogenies used an amino-acid percentage distance substitution model and 1,000 bootstrap reiterations. All sequences were submitted to GenBank under accession nos. HM368166–HM368175. All analyses were performed with Epi Info 3.5.1 (www.cdc.gov/epiinfo) and with SPSS 17 (SPSS, Munich, Germany).

## Results

**Figure 2:**
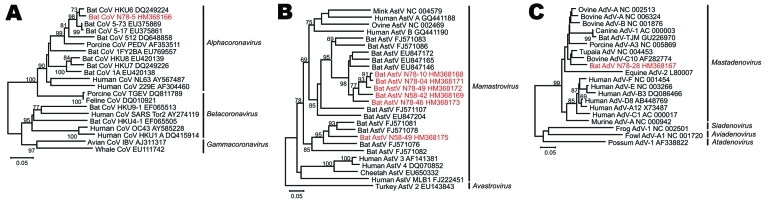
Phylogenetic relationships of novel bat viruses. A) Coronavirus, B) astrovirus, C) adenovirus. Neighbor-joining phylogenies were generated with MEGA (www.megasoftware.net), by using an amino acid percentage distance substitution model drawn to scale, complete deletion option, and 1,000 bootstrap reiterations. Bootstrap values are shown next to the branches; values <65 were removed for graphic reasons. Viruses newly identified in this study are shown in red. Viral genera are depicted next to taxon names. The BLOSUM aligned datasets corresponded to an 816-nt alignment, corresponding to nucleotides 14,781–15,596 in severe acute respiratory syndrome coronavirus (SARS-CoV) strain Tor2 (GenBank accession no. AY274119) for CoVs (A); a 381-nt alignment corresponding to nt 3,437–3,817 in mink astrovirus (AstV) (GenBank NC_004579) for AstVs (B); and to a 255-nt alignment corresponding to nt 46–300 in the bovine adenovirus (AdV) C10 hexon gene (GenBank accession no. AF282774) for AdVs (C). Trees were visualized in MEGA4, with prototype virus sequences restricted to ≈20 taxa additional to newly identified viruses for graphic reasons. Scale bars indicate amino acid substitutions per site.

In a first step, the *M. myotis* maternity colony was surveyed for bat-borne RNA viruses. Broad-range RT-PCR assays for CoVs and AstVs were employed on samples taken in 2008. Screening was extended to include AdVs described in microchiroptera and megachiroptera bats (*6**,**8**,**20*). As shown in Figure 2, a CoV, 6 different AstVs, and 1 novel AdV were found. The CoV (GenBank accession no. HM368166) was a member of the genus *Alphacoronavirus* and belonged to a tentative species defined by bat-CoV HKU6 (97.4% amino acid identity in RNA-dependent RNA polymerase [RdRp], typing criteria as defined in [*9*]). The 6 different mamastroviruses (GenBank accession nos. HM368168–HM368175) clustered phylogenetically with bat-associated AstV, which has been described previously (*4**,**21*), showing 65.0%–86.0% amino acid identities with related bat-associated AstV from *M. chinensis* and *M. ricketti* bats from the People’s Republic of China (Figure 2). The AdV constituted a novel *Mastadenovirus* species (GenBank accession no. HM368167) that was clearly separated from a clade of AdV recently reported in a *M. ricketti* bat in China and a *Pipistrellus pipistrellus* bat in Germany (*6**,**20*) (A. Kurth, pers. comm.). The closest relatives were bovine AdV C10 (GenBank accession no. AF282774) and Tupaia AdV (GenBank accession no. NC_004453), with 90.0% and 91.0% identity on the amino acid level, respectively. Amino acid identity with the Chinese bat AdV TJM (GenBank accession no. GU226970) was 83.5%.

**Figure 3:**
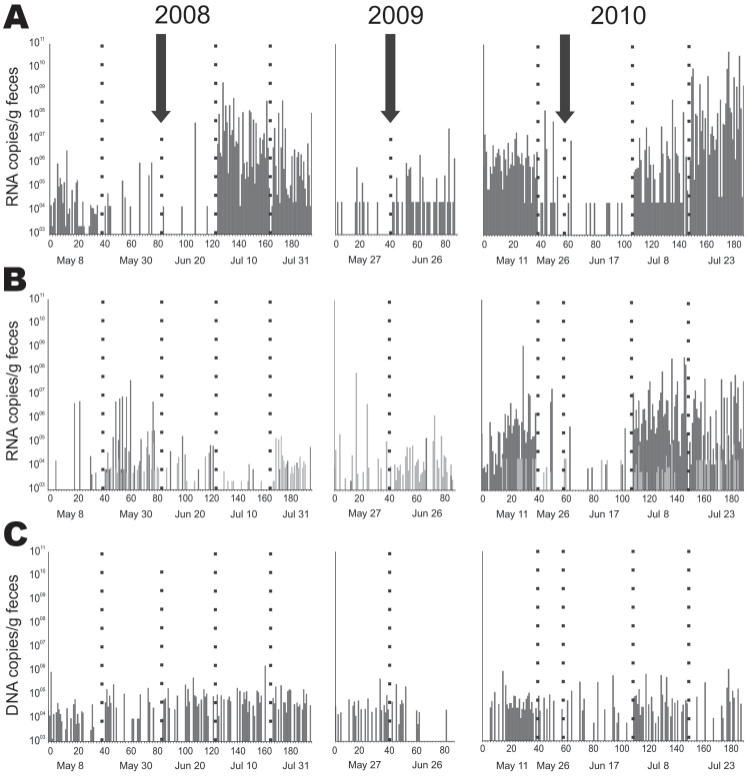
Detection frequency of bat viruses and virus nucleic acid concentrations over time. A) Coronavirus; B) astrovirus; C) adenovirus. Samples were obtained approximately every 3 weeks from the same *Myotis myotis* bat maternity roost in 3 different sampling years, 2008–2010. Each sample was tested by specific real-time reverse transcription–PCR (RT-PCR) with RNA/DNA concentrations per gram of feces given on the y axis. The arrows indicate the time of birth of the first pup. Numbers on the x-axis represent individual fecal pools tested, consisting of 5 single fecal pellets each. Five different sampling dates (below each panel) are shown by dotted lines for each sampling year. Empty columns indicate pools that tested negative. In panel B, light and dark gray bars identify results by 2 different real-time RT-PCRs that were used simultaneously to cover the large astrovirus diversity encountered.

For all 3 viruses, strain-specific real-time RT-PCR assays, including cloned, in vitro–transcribed RNA or plasmid DNA quantification standards, were generated (Table 1). For AstV, 2 assays had to be designed to cover the high diversity of AstVs that was found. These assays were used to monitor virus abundance in the *M. myotis* bat maternity colony over time. Populating of the roost started in March 2008. Sampling started in the second week of May when the colony reached full size. Sampling extended over 5 sampling dates until late July 2008 (Figure 3); 195 pooled samples, equal to 975 fecal pellets, were collected during this time. As shown in Figure 3, panel A, 2 peaks of amplification of CoV occurred, characterized by increased virus concentrations and increased detection rates. The first peak was observed in the first sample taken after populating of the roost. In this sample, 77.5% of specimens contained virus, whereas the succeeding 2 samples showed a statistically significant 2- to 8-fold decrease of detection frequency (χ^2^ 43.4, p<0.001). A second and more significant amplification occurred ≈1 month after parturition, with 100% of collected fecal samples testing positive for CoV RNA (sampling dates 4 and 5). The second peak was characterized by an increase in median RNA concentration by ≈2 orders of magnitude (Table 2). Peak concentration was 2,453,390,770 CoV RNA copies/g of feces. The increased virus detection rate in the post-parturition period in comparison to the preceding 2 sampling dates, as well as the observed increase in virus concentration were statistically highly significant (analysis of variance [ANOVA], *F* = 24.7, p<0.001; χ^2^ 107.9, p<0.001).

**Table 2:** Viral RNA detection characteristics over time, Germany, 2008–2010*

Sampling date	No. fecal pellets	Coronavirus				Astrovirus				Adenovirus		
		No. (%) positive	Log abun	Log conc		No. (%) positive	Log abun	Log conc		No. (%) positive	Log abun	Log conc
2008 May 8	40	31 (77.5)	4.13	4.24		11 (27.5)	3.10	3.66		23 (57.5)	3.93	4.17
2008 May 30	44	10 (22.7)	4.01	4.65		32 (72.7)	4.39	4.53		16 (36.4)	4.21	4.65
2008 Jun 20	40	4 (10.0)	3.19	4.19		13 (32.5)	3.92	4.41		22 (55.0)	4.61	4.87
2008 Jul 10	40	40 (100.0)	6.88	6.88		9 (22.5)	2.73	3.37		22 (55.0)	4.59	4.85
2008 Jul 31	31	31 (100.0)	5.76	5.76		22 (71.0)	3.93	4.08		20 (64.5)	4.45	4.64
2009 May 27	40	9 (22.5)	3.73	4.38		14 (35.0)	3.72	4.18		19 (47.5)	4.10	4.42
2009 Jun 26	48	35 (72.9)	4.24	4.38		32 (66.7)	3.90	4.07		11 (22.9)	3.93	4.57
2010 May 11	40	40 (100.0)	6.21	6.21		39 (97.5)	5.41	5.42		27 (67.5)	4.42	4.59
2010 May 26	18	9 (50.0)	4.07	4.37		4 (22.2)	5.75	6.41		7 (38.9)	4.29	4.70
2010 Jun 17	49	10 (20.4)	3.66	4.35		11 (22.4)	3.62	4.26		13 (26.5)	3.85	4.42
2010 Jul 8	40	39 (97.5)	5.79	5.80		40 (100.0)	6.31	6.31		27 (67.5)	4.44	4.61
2010 Jul 23	40	39 (97.5)	7.91	7.92		39 (97.5)	5.58	5.59		12 (30.0)	4.32	4.84

*Abun, median virus concentration multiplied by prevalence; conc, median virus concentration, RNA/DNA copies per gram of feces in positive samples.

For AstV, no amplification was associated with parturition in the same samples. Total detection rate of astroviruses was 51.2% before birth of the first pup and 40.5% thereafter. However, prevalence and virus concentration significantly increased in the second sampling than in the first and fourth samplings, respectively (χ^2^ 7.4, p = 0.006); ANOVA, *F* = 4.4, p = 0.03). This pattern resembled the amplification after formation of the colony as also observed in CoV. Figure 3, panel B, shows AstV RNA concentrations over time.

Concentration and detection rates of AdV were determined next. As shown in Figure 3, panel C, no marked variation in prevalence was seen. Detection rate was 46.4% before birth of the first pup and 57.7% thereafter. Although statistically significant variation in virus concentrations could be observed (ANOVA, *F* = 8.2, p<0.001), this was exclusively contributed by slightly lower virus concentrations in the first sampling than in the succeeding samples (Table 2).

Because of the diverging pattern of amplification of the RNA viruses (CoVs, AstVs) against the DNA virus (AdV), the investigation was repeated the next year (2009). All viruses were detected again (Figure 3). Unfortunately, the colony was found to be abandoned after the first postparturition sampling, leaving an incomplete dataset for that year. Still, it could be seen and statistically confirmed that the CoV was beginning to be amplified after parturition (χ^2^ 7.85, p = 0.005), while no significant variation in prevalence or virus concentration was visible for the other viruses (data not shown).

A repetition of the full sampling scheme was attempted again in 2010. All 5 sampling dates could be completed, yielding a sample of 187 pools in total, equivalent to 935 individual fecal pellets. As shown in Figure 3, the CoV showed the same 2 amplification peaks as observed in 2008, one after formation of the colony and one after parturition. Mean virus concentrations these samples were significantly increased compared with the samples taken at other times (ANOVA, *F* = 22.0, p<0.001). The detection rate during the first peak was 100.0%, followed by 2-fold and 5-fold decreases 3 and 6 weeks later (χ^2^ 52.0, p<0.001), and an augmentation to 97.5% after parturition (χ^2^ 77.7, p<0.001). The maximal CoV concentration in 2010 was higher than in 2008, at 50,495,886,830 RNA copies/g of feces. The amplification pattern of AstV showed clearer similarities to that of CoV in 2010. An initial peak of detection rate was 97.5%, followed by a detection rate of 22.2%–22.4% in subsequent samples and 97.5%–100% after parturition (χ^2^ 56.2 and 92.2, respectively, p<0.001). Virus concentrations were significantly increased in these amplification peaks (ANOVA, *F* = 7.8, p<0.001). The amplification was almost completely contributed by one of the AstV lineages (represented by BtAstV/N58–49), while the other lineages were constant (Figure 3, panel B). Notably, the BtAstV/N58–49 lineage had been present only sporadically in the years before (Figure 3, panel B). Detection frequency for AdV was 58.6% before parturition and 40.3% thereafter without any significant variation in virus concentrations between sampling dates (ANOVA, *F* = 0.5, p = 0.72).

### Effect of Virus Abundance of Bat Reproductive Success

**Figure 4:**
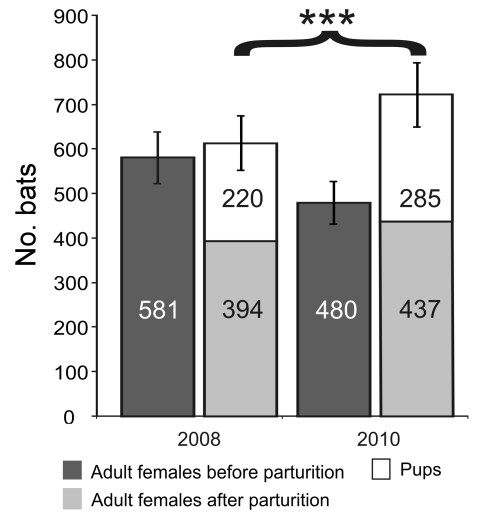
*Myotis myotis* bat maternity roost composition and reproductive success. Age composition of bats composing the *M. myotis* maternity roost under study are depicted before and after parturition in 2 different sampling years, 2008 and 2010. The y-axis represents the number of individual bats, additionally indicated in individual bars. The brace and asterisks represent statistical significance of the gain in total colony size after parturition in 2010, compared with colony size in 2008. Error bars represent an assumed 10% error margin in counting.

CoV, AstV, and AdV are clearly pathogenic for other mammals. To determine whether the presence of these viruses had any influence on bats’ health, the reproductive success of the maternity colony was evaluated in 2008 and 2010. The data are summarized in Figure 4. In a census taken 2008 before parturition, the colony comprised 581 female adult bats. A second census after parturition yielded 394 adults and 220 newborns. The decline in adult females and the moderate number of pups contrasted with observations made in 2010, when 480 adult females were counted before parturition and 437 thereafter, along with 285 pups. The gain in total colony size was significantly greater in 2010 than in 2008 (χ^2^ 18.3, p<0.001).

## Discussion

Viral host switching is probably determined by the chances of interspecies contact, as well as by the concentration and prevalence of virus in the donor species. To judge zoonotic risks associated with bats, when and where these 2 variables would favor transmission must be determined. In this study, we found that strong and specific amplification of the RNA viruses, but not of the DNA virus, occurred upon colony formation and following parturition.

The viruses monitored in our study were selected because they are regularly encountered in bats and thus provide a certain chance of detection. Attempts to characterize virus dynamics in bat populations have been made earlier by using the examples of lyssaviruses (rabies virus and related species), filoviruses (Ebola and Marburg viruses) and henipaviruses (Hendra and Nipah viruses). However, because these viruses are found rarely, only vague conclusions have so far been made. For instance, increased contact between bats and humans through bat migratory events or fruit harvesting periods have been temporally linked with individual human cases of Ebola and Nipah virus infection (*22**,**23*). One study has shown that the success of Nipah virus isolation from *Pteropus* spp. bats depended on seasonal factors, which was interpreted as evidence for season-dependent variation of virus concentration or prevalence (*24*). Furthermore, the reproductive cycle of bats has been tentatively connected with seasonality of henipavirus, filovirus and lyssavirus seropositivity in bats as well as with the temporal distribution of Nipah virus outbreaks in humans (*10**,**12**,**15**,**25**–**27*). Our direct data on virus concentration and prevalence for CoV and AstV integrate many of these independent observations and provide a model that might be transferable to other viruses. The initial peak in annual CoV and AstV prevalence observed in our study was probably due to the formation of a contiguous population of sufficient size and density, bringing together enough susceptible bats to establish a critical basic reproductive rate of infection (*28**,**29*). The second amplification peak after parturition was most probably associated with the establishment of a susceptible subpopulation of newborn bats who had not yet mounted their own adaptive immunity. Sporadic vertical transmission from mothers to pups as observed in *Pteropus* spp. bats artificially infected with Hendra virus would probably initiate this second wave of infection (*30*). The main driver of the second wave would then be a horizontal transmission between pups. The latency between parturition and the second wave of virus amplification indicates a certain level of perinatal protection conferred by mothers during the first weeks of life as demonstrated for other small mammals, and as indirectly suggested for bats (*13**,**31**–**33*). This protection may be differentially effective against different viruses, as indicated by the differential amplification patterns between CoVs and AstVs. While CoVs were amplified both in 2008 and 2010, AstVs underwent postparturition amplification only in 2010 when a new virus lineage gained predominance in the population. This finding strongly indicates antigen-specific immune control of virus circulation.

A common, but unproven, assumption is that bats are resistant to even highly pathogenic viruses (*2**,**3*). In this study, we have correlated direct measurements of virus burden with the reproductive success of a bat colony. The rate of successful reproduction is probably a sensitive indicator of the presence or absence of disease, given the tenuous conditions under which bats breed in temperate climates. Indeed, no effects of CoV and AstV on reproduction were initially apparent; although postparturition amplification of both viruses was more efficient in 2010 than in 2008, the overall breeding success was significantly better in 2010. This result may merely have been a consequence of a positive correlation between virus amplification and colony size, which was larger in 2010 due to better breeding success. On the other hand, the individual prenatal amplification peaks of both CoV and AstV were higher in 2010, which may have enabled better perinatal protection and thus better survival of newborns. The grouping of large numbers of pregnant females before birth is a specific characteristic of bats that may contribute to their puzzling ability to maintain highly pathogenic viruses without experiencing die-offs. Our noninvasive approach did not allow any further analyses such as the testing of blood and colostrum samples for antibodies. Nevertheless, the general picture obtained in this study by correlating virus and bat population dynamics suggests that bats control infections in similar ways to other mammals, and that they may well experience virus epidemics.

Another intriguing finding of our study was the difference in the amplification pattern of the RNA viruses and that of the DNA virus. We selected these viruses because, in humans, AdVs are typically capable of persisting in tissue (*34*) and thus do not depend so much on continuous transmission and consistent amplification on the population level. Indeed, it appeared that AdV did not make use of periodic amplification in our bat colony. Persistence on the level of individual bats is more common for DNA viruses than for RNA viruses. RNA viruses ensure that they are maintained on a population level by a much higher error rate of the enzymes they use for genome replication and consequent higher levels of antigenic variability, causing waves of epidemic spread as confirmed for bat-borne RNA viruses in this study. This factor can explain why most emerging viruses, including those from bats, are indeed RNA viruses (*2**,**35*).

For CoV, our study indicates clearly that virus amplification takes place in maternity colonies, confirming our earlier statistical implications from studies in a different region and on a different species (*16*). High peak RNA concentrations in the range of 10^9^–10^10^ copies/g were observed, which is tremendously higher than CoV concentrations observed in earlier studies outside the parturition period (*19*). Similarly high RNA virus concentrations are observed in human diseases transmitted through the fecal-oral route, e.g., picornaviruses or noroviruses, which suggests that maternity roosts may involve an elevated risk of virus transmission to other hosts. It is interesting to reconsider the potential genesis of the SARS epidemic in this light. Although an origin of SARS-related CoV in bats is confirmed (*9**,**36*), SARS-CoV precursors have existed in carnivores some time before the SARS epidemic and have been transmitted from carnivores to humans again at least one additional time after the end of the epidemic (*37**,**38*). these data provide an intriguing explanation of how the SARS agent may have left its original reservoir (*39**,**40*). The data also indicate a feasible and ecologically sensible means of prevention. Because carnivores are known to enter maternity roosts to feed on dead newborn bats, bat maternity roosts should be left undisturbed by humans and kept inaccessible to domestic cats and dogs.
